# miR-106b-5p promotes renal cell carcinoma aggressiveness and stem-cell-like phenotype by activating Wnt/β-catenin signalling

**DOI:** 10.18632/oncotarget.15591

**Published:** 2017-02-21

**Authors:** Jun Lu, Jin-Huan Wei, Zi-Hao Feng, Zhen-Hua Chen, Yong-Qian Wang, Yong Huang, Yong Fang, Yan-Ping Liang, Jun-Jie Cen, Yi-Hui Pan, Bing Liao, Wen-Fang Chen, Wei Chen, Jun-Hang Luo

**Affiliations:** ^1^ Department of Urology, First Affiliated Hospital, Sun Yat-sen University, Guangdong, China; ^2^ Department of Musculoskeletal Oncology, First Affiliated Hospital, Sun Yat-sen University, Guangdong, China; ^3^ Department of Pathology, First Affiliated Hospital, Sun Yat-sen University, Guangdong, China

**Keywords:** miR-106b-5p, stemness, Wnt signalling, tumorigenesis, renal cell carcinoma

## Abstract

**Purpose:**

To examine the role of miR-106b-5p in regulating the cancer stem-cell-like phenotype in clear cell renal cell carcinomas (ccRCC).

**Experimental Design:**

Real-time PCR was performed to evaluate miR-106b-5p levels in ccRCC cell lines and patients specimens. A series of *in vivo* and *in vitro* assays were performed to confirm the effect of miR-106b-5p on ccRCC stemness phenotype.

**Results:**

ccRCC cells and tissues expressed more miR-106b-5p than normal controls. Gain- and loss-of-function studies demonstrated that overexpression of miR-106b-5p in ccRCC cells increased the spheres formation ability and the proportion of side population cells. Ectopic expression of miR-106b-5p in ccRCC cells increased tumour growth rates and the number of metastatic colonies in the lungs by using an orthotopic kidney cancer model and a tail vein injection model, respectively. Mechanistic studies revealed that, miR-106b-5p has an activating effect on Wnt/β-catenin signalling. miR-106p-5p overexpression simultaneously targets multiple negative regulators of the Wnt/β-catenin pathway, namely, LZTFL1, SFRP1 and DKK2. In addition, we also confirmed that miR-106b-5p and its targets expression correlates with the overall-survival of ccRCC patients from TCGA.

**Conclusions:**

These findings suggest that miR-106b-5p mediates the constitutive activation of Wnt/β-catenin signalling, likely serving as a potential therapeutic target for ccRCC.

## INTRODUCTION

Renal cell carcinomas (RCC) is one of the most common malignant solid tumors in human, and the incidence and mortality of RCC are on the rise every year [1]. RCC are classified into different pathological subtypes, and clear cell RCC account for 70% to 80%. The prognosis of metastatic ccRCC patients remains generally dismal and its 5-year survival rate is ∼10 percent [2]. Surgical resection is the standard of care for localized RCC, but about 30–40% of the patients may experience disease progression or relapse after initial radical surgery [3–5]. The majority of ccRCC treatment failures are due to resistance to both traditional chemotherapy and radiation. Hence, it would be helpful to the discovery of new therapy strategies by exploring the molecular mechanisms underlying the development and progression of ccRCC.

Methylation of CpG nucleotides [6] or genes mutation [7–9] are associated with Wnt signalling activation and ccRCC malignancy. These findings suggest that Wnt signaling pathway may have important prognostic and therapeutic value in ccRCC. The knockdown of several Wnt signalling antagonists, like WIF1, Dkk, SFRP1, SOSTDC1 and IGFBP4 have been reported to play important roles in the aberrant Wnt signaling in ccRCC [10–13]. Wnt antagonists are critical in the pathogenesis of ccRCC, but the molecular mechanism of loss of function of Wnt antagonists in ccRCC remains unclear. Wnt antagonists play critical role in the pathogenesis of ccRCC, but the molecular mechanisms of the down regulation of Wnt antagonists in ccRCC is still unclear.

It has been found that different miRNAs modulate the Wnt signalling in human malignancy tumors [14]. miR-106b-5p belongs to the microRNA precursor miR-17 family, and there have been a number of studies based on its roles in tumor proliferation and metastasis [15–19]. Until now, the role of miR-106b-5p in regulating the cancer stem-cell-like phenotype in ccRCC remains uncertain.

In this study, we found that miR-106b-5p overexpression promotes ccRCC cells stemness both *in vitro* and *in vivo*. miR-106b-5p plays important role in the down regulation of Wnt antagonists in ccRCC by simultaneously targets LZTFL1, SFRP1 and DKK2. Furthermore, miR-106b-5p and its targets are closely associated with ccRCC patient survival by analysing TCGA data. Our data show miR-106b-5p plays important roles in the sustainment of ccRCC cells stemness.

## RESULTS

### Expression levels of miR-106b-5p in ccRCC cells and tissues

**Figure 1:**
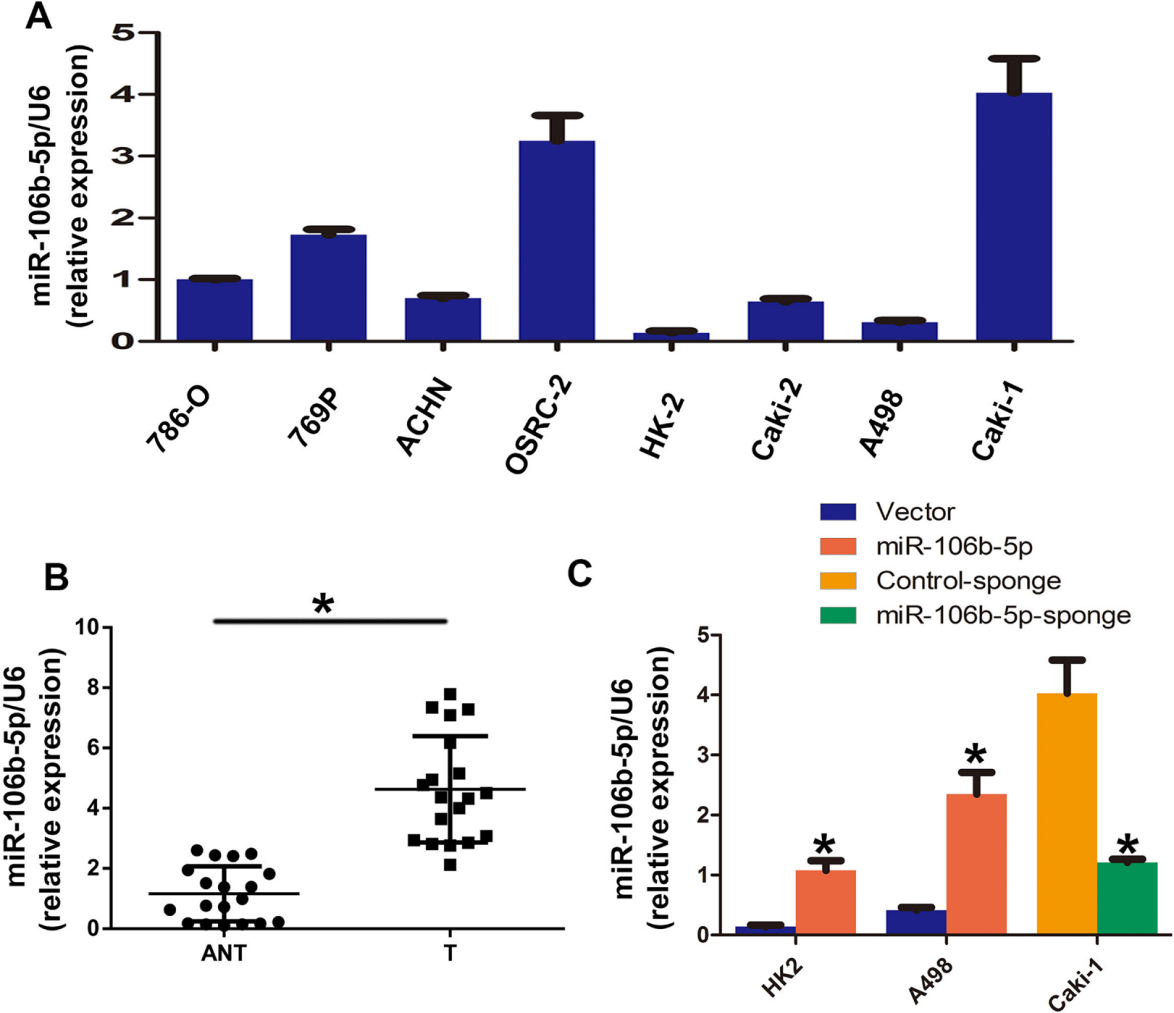
miR-106b-5p is overexpressed in ccRCC cell lines and tissues (**A**) RT–qPCR analysis of miR-106b-5p expression in ccRCC Cell lines, including an immortalized proximal tubule epithelial cell line HK2 and a panel of seven human ccRCC cell lines. (**B**) Relative expression of miR-106b-5p in 20 pairs of ccRCC tumour tissues (T) and their corresponding adjacent non-cancerous tissues (ANT). (**C**) Relative expression of miR-106b-5p expression in the indicated cell lines using real-time PCR. Each bar represents the mean ± s.e.m. derived from three independent experiments. A two-tailed Student's *t*-test was used for statistical analysis (*P < 0.05).

In this study, we first used real-time PCR to examine the expression levels of miR-106b-5p in eight ccRCC cells and 20 pairs of ccRCC and adjacent normal tissues. The results showed that all seven renal cell carcinoma cell lines (786-O, A498, ACHN, OSRC-2, Caki-2, 769-P, Caki-1) had higher levels of miR-106b-5p expression than that in the normal kidney line HK-2. In addition, the metastatic ccRCC cell line Caki-1 showed the highest levels of miR-106b-5p (Figure 1A). As shown in Figure 1B, real-time PCR results shown significantly up-regulation of miR-106b-5p in tumor tissues than normal tissues (*p* < 0.0001, paired *t*-test). We next silenced miR-106b-5p in Caki-1 cells and overexpressed miR-106b-5p in HK2 and A498 cells. As shown in Figure 1C, the relative miR-106b-5p levels were obviously upregulated in miR-106b-5ptransduced HK2 and A498 cells compared to the control cells (*p* < 0.0001, paired *t*-test). In addition, miR-106b-5p-sponge transduction significantly decreased the miR-106b-5p levels in Caki-1 cells (*p* < 0.0001, paired *t*-test).

### Regulation stemness of miR-106b-5p in ccRCC cells

**Figure 2:**
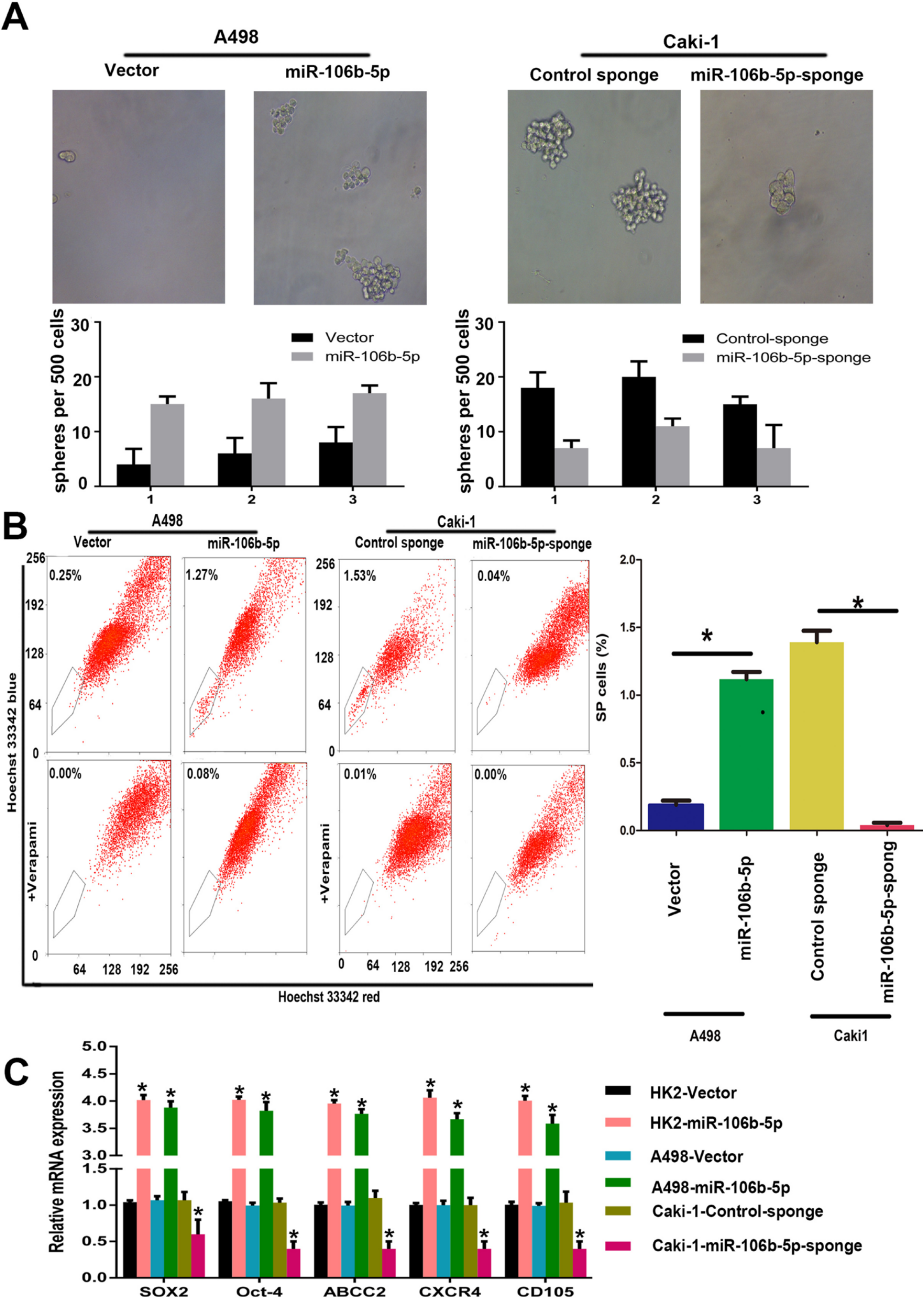
miR-106b-5p promotes stem cell-like properties in ccRCC cells *in vitro* (**A**) Representative images and quantification of spheres formed by the indicated cells. (**B**) Hoechst 33342 dye exclusion assay showing that overexpressing miR-106b-5p increased the SP cell proportions in the indicated cells, whereas silencing miR-106b-5p decreased these proportions. (**C**) RT–qPCR analysis of the expression levels of cancer stemness associated markers, including SOX2, OCT4, ABCG2, CD105, and CXCR4 in miR-106b-5p-overexpressing and miR-106b-5p-silenced cells compared with the corresponding control cells. Each bar represents the mean±s.e.m derived from three independent experiments. A two-tailed Student's *t*-test was used for statistical analysis (*P < 0.05).

To see if miR-106b plays roles in the regulation of ccRCC cells stemness phenotype, we next examined the impacts of miR-106b-5p on ccRCC cells self-renewal ability. We can see that the size and numbers of spheres increased in miR-106b-5p overexpression A498 cells and decreased in miR-106b-5p knockdown Caki-1 cells (Figure 2A). In addition, more side population (SP) cells were seen in miR-106b-5p-transduced- A498 cells than vector control cells, whereas fewer SP cells were seen in miR-106b-5p-silenced Caki-1 cells compared with control cells (Figure 2B). Notably, overexpression of miR-106b-5p in A498 and HK-2 cells leads to upregulation of ccRCC stemness associated markers like Sox2, Oct4, ABCC2, CXCR4 and CD105, and the results were opposite in miR-106b-5p-silenced cells (Figure 2C). Our data indicate that miR-106b-5p promotes ccRCC cells stemness *in vitro*.

### Impact of miR-106b-5p on tumor growth and metastasis of ccRCC cells *in vivo*

**Figure 3:**
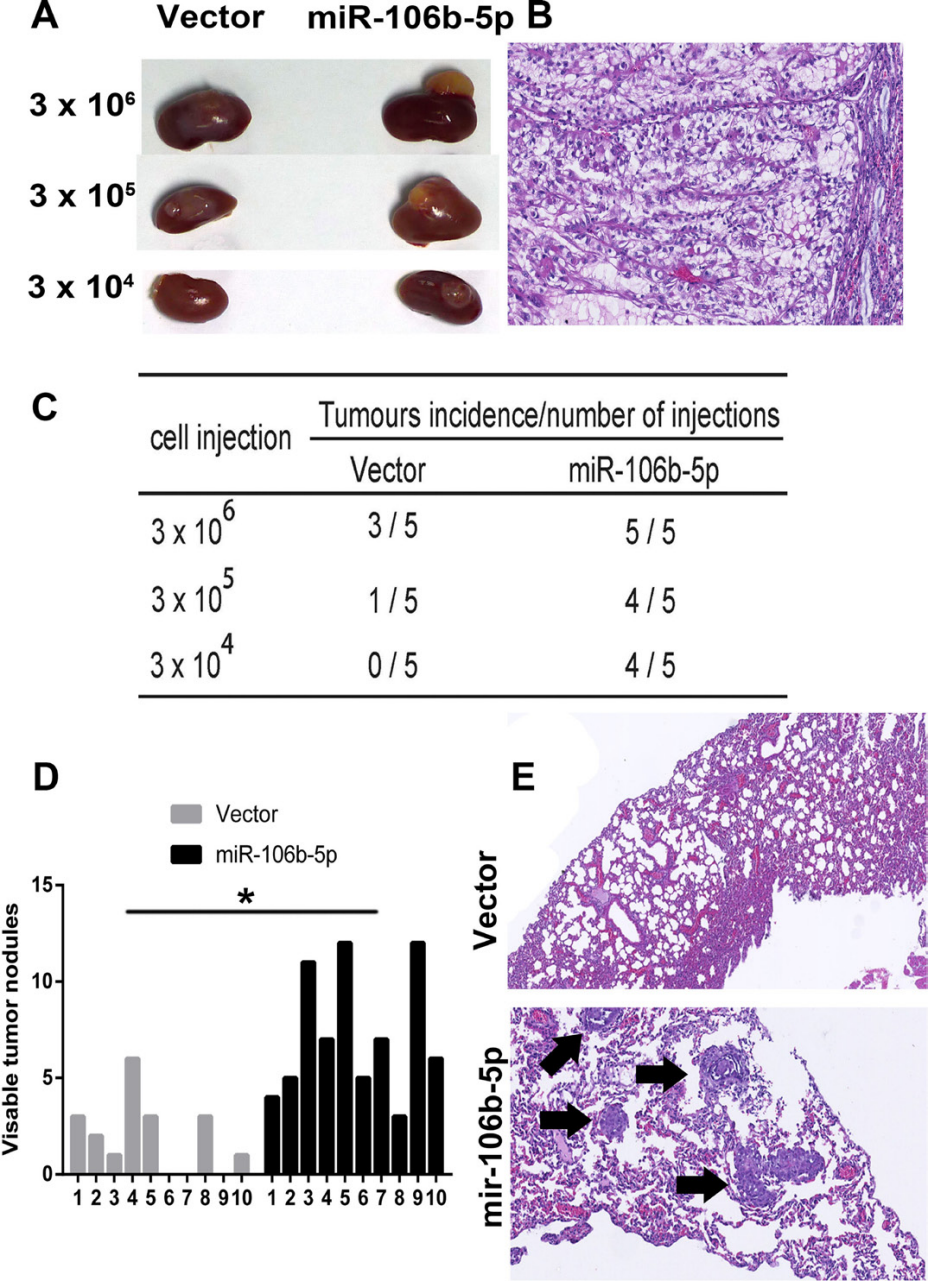
miR-106b-5p enhances the tumorigenicity and metastasis of ccRCC cells *in vivo* (**A**) In total, 3 × 10^6^, 3 × 10^5^ and 3 × 10^4^ Caki-1-vector and Caki-1-miR-106b-5p-transfected cells mixed with Matrigel were implanted in the renal capsules of BALB/c nude mice (*n* = 5 per group). Representative images of the tumours are shown. (**B**) Representative image of H&E staining showing orthotopic xenograft tumors in the mouse kidney. (**C**) Tumour formation frequencies in orthotopic xenograft tumor model for different numbers of the indicated cells are shown (**D**) The number of nodules was qualified on lungs of BALB/c nude mice (*n* = 10 per group) 6 weeks after tail vein injection of A498-vector cells and A498-miR-106b-5p. (**E**) Representative metastatic lesions stained by H&E in the lungs of mice. Arrows denotes the metastatic colonies in the lung tissues. The data are presented as the mean ± s.e.m. A two-tailed Student's *t*-test was used for statistical analysis (*P < 0.05).

We further studied the *in vivo* impact of miR-106b-5p on ccRCC cell growth and metastasis by injecting A498 cells containing either a control or miR-106b-5p-overexpression vector into BALB/c nude mice, either orthotopically or via the tail vein. To assess the role of miR-106b-5p in ccRCC cells tumorigenicity, different doses (3 × 10^6^, 3 × 10^5^ and 3 × 10^4^) of miR-106b-5p transduced cells and control cells were orthotopically implanted into renal capsules of the mice. As shown in Figure 3A–3C, miR-106–5p overexpression in ccRCC cells promoted tumorigenesis and increased tumour growth rate. In the mouse metastasis model, the numbers of metastatic colonies in the lungs were dramatically increased in mice injected with miR-106b-5p-overexpressing cells, compared to control cells (Figure 3D, 3E).

### The role of miR-106b-5p in activating Wnt signalling to promote ccRCC cells stemness

**Figure 4:**
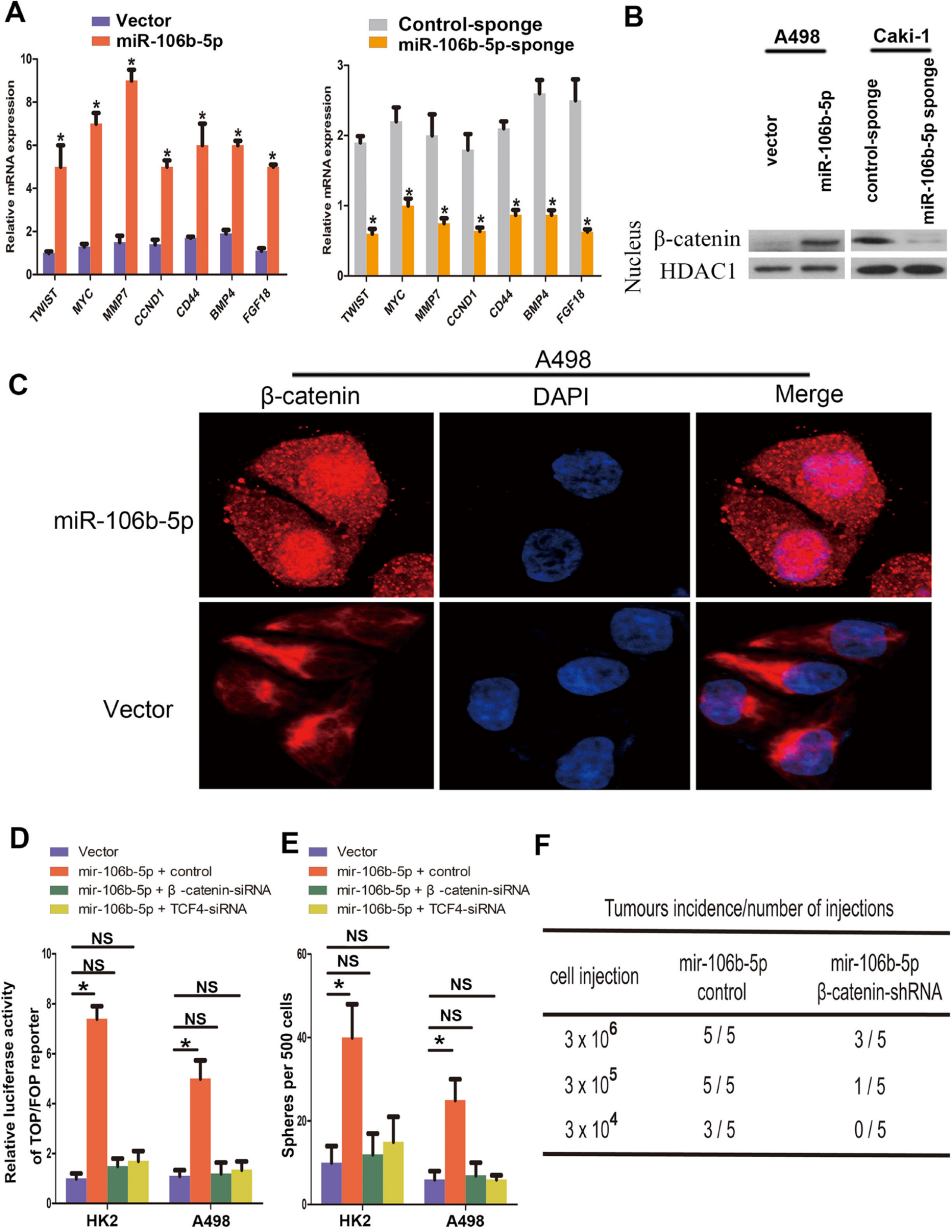
miR-106b-5p activates Wnt/β-catenin signalling (**A**) RT–qPCR analysis of the expression of the established downstream targets for the Wnt/β-catenin pathway, including TWIST, MYC, MMP7, CCND1, CD44, BMP4 and FGF18, in the indicated cells. (**B**) Altered nuclear translocation of β-catenin in response to ectopic miR-106b-5p expression. Nuclear fractions of the indicated cells were analysed by western blot analysis. HDAC1 was used as a loading control. (**C**) Subcellular β-catenin localization in the indicated cells was assessed by immunofluorescence staining. (**D**) Luciferase assay of TCF/LEF transcriptional activity in indicated cells. (**E**) Representative images and quantification of cellular spheres formed by the indicated cells. (**F**) Tumour formation frequencies for the different numbers of indicated cells. Each bar represents the mean ± s.e.m. derived from three independent experiments. A two-tailed Student's *t*-test was used for statistical analysis (*P < 0.05, NS: not statistically significant).

Since the Wnt signalling pathway plays critical role in maintaining cancer stemness, we next examined the impact of miR-106b-5p overexpression on Wnt signalling. miR-106b-5p overexpression in A498 cells significantly increased the mRNA expression levels of seven well-known genes relevant to Wnt/β-catenin pathway (Figure 4A). miR-106b-5p overexpression in ccRCC cells enhanced the nucleus translocation of β-catenin as determined by western blotting analysis of cellular fractionation, and the results were reverse in miR-106b-5p knockdown Caki-1 cells (Figure 4B). Meanwhile, immunofluorescence analysis showed that overexpression of miR-106b-5p in A498 cells increased nuclear accumulation of β-catenin (Figure 4C). We then evaluated the elimination of Wnt signalling on miR-106b-5p mediated cancer cell stemness. As we shown in Figure 4D–4F, β-catenin or TCF4 knockdown by siRNA transfection directly repressed Wnt signalling, eliminated the enhancement of miR-106b-5p overexpression on ccRCC cells stemness as determined by spheres formation assay and *in vivo* assay. These results indicate that miR-106b-5p promote stemness and tumorigenesis in ccRCC cells is dependent on the activating of Wnt signalling.

### The regulation of miR-106b-5p on LZTFL1, SFRP1 and DKK2

**Figure 5:**
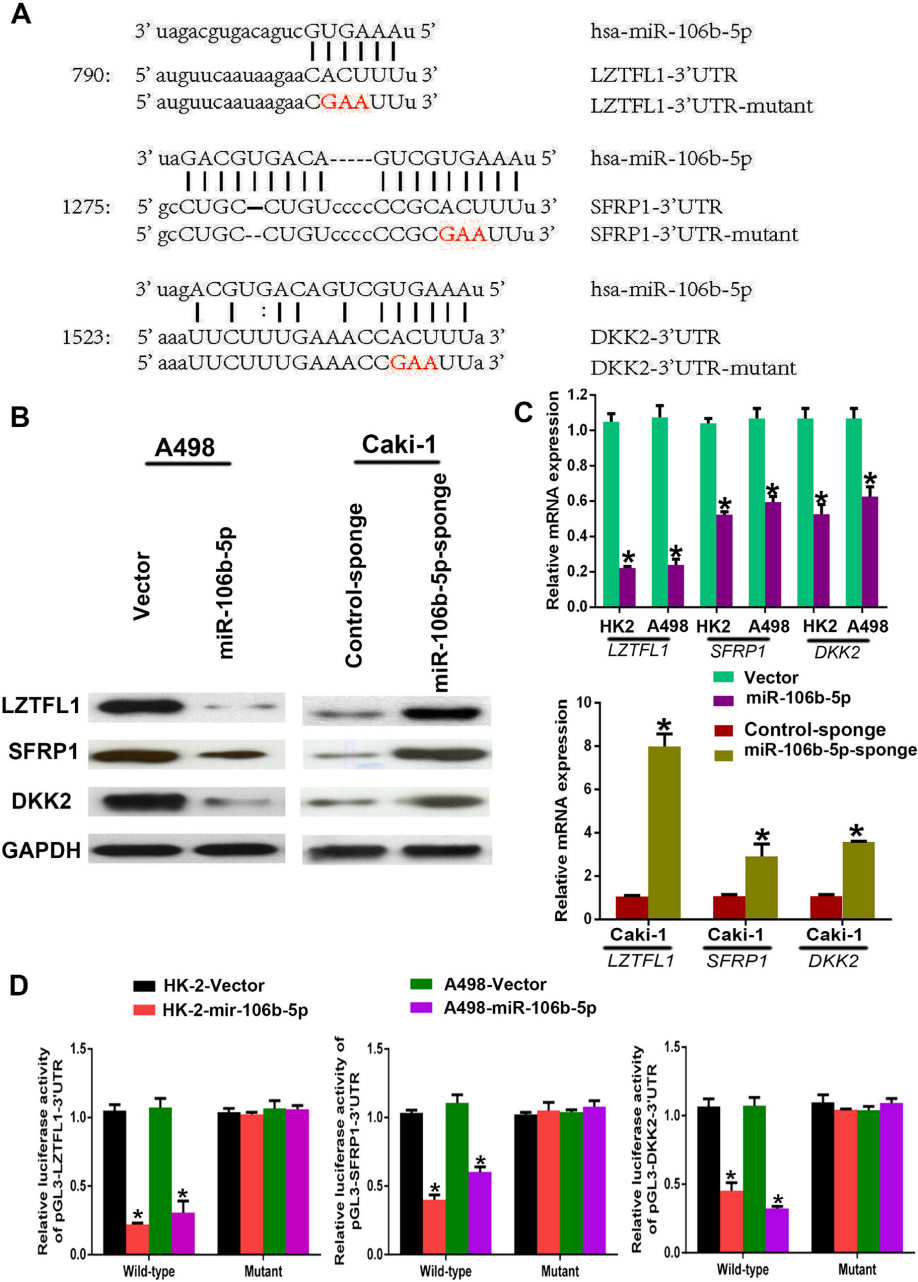
miR-106b-5p directly targets multiple negative regulators of the Wnt pathway (**A**) Predicted binding sites of miR-106b-5p in the wild type 3′-UTRs of LZTFL1, SFRP1 and DKK2. Mutations in these 3′-UTRs are highlighted in red. (**B**, **C**) Western blot and RT–qPCR analyses of LZTFL1, SFRP1 and DKK2 expression in the indicated cells. (**D**) Luciferase activity of reporters with wild type or mutant 3′-UTRs of LZTFL1, SFRP1 and DKK2 in the indicated cells co-transfected with the indicated oligonucleotides. Each bar represents the mean ± s.e.m. derived from three independent experiments. A two-tailed Student's *t*-test was used for statistical analysis (*P < 0.05).

To explore the potential mechanisms underlying miR-106b-5p activating Wnt signalling in ccRCC cells, we first used miRanda and TargetScan databases to mine miRNA targets. We then found three well-known Wnt signalling antagonists, LZTFL1, SFRP1 and DKK2, which may be inhibited by miR-106b-5p (Figure 5A). Furthermore, the mRNA and protein levels of LZTFL1, SFRP1 and DKK2 were significantly down-regulated in miR-106b-5p overexpression ccRCC cells but up-regulated in miR-106b-5p-silenced cells as determined by qPCR and western blotting, respectively (Figure 5B, 5C). Subsequently, luciferase reporter assay was used to confirm the targeting of miR-106b-5p to the 3′ untranslated regions (3′-UTRs) of LZTFL1, SFRP1 and DKK2. As shown in Figure 5D, the luciferase activity was reduced in miR-106b-5p overexpression HK-2 and A498 cells. However, the suppression ability of miR-106b-5p overexpression on LZTFL1, SFRP1 and DKK2 were eliminated when the predicted miR-106b-5p target sites were mutation. These data suggest that miR-106b-5p simultaneous suppressed LZTFL1, SFRP1 and DKK2 by binding to there 3′-UTRs.

### The prognostic value of miR-106b-5p and its targets in ccRCC

**Figure 6:**
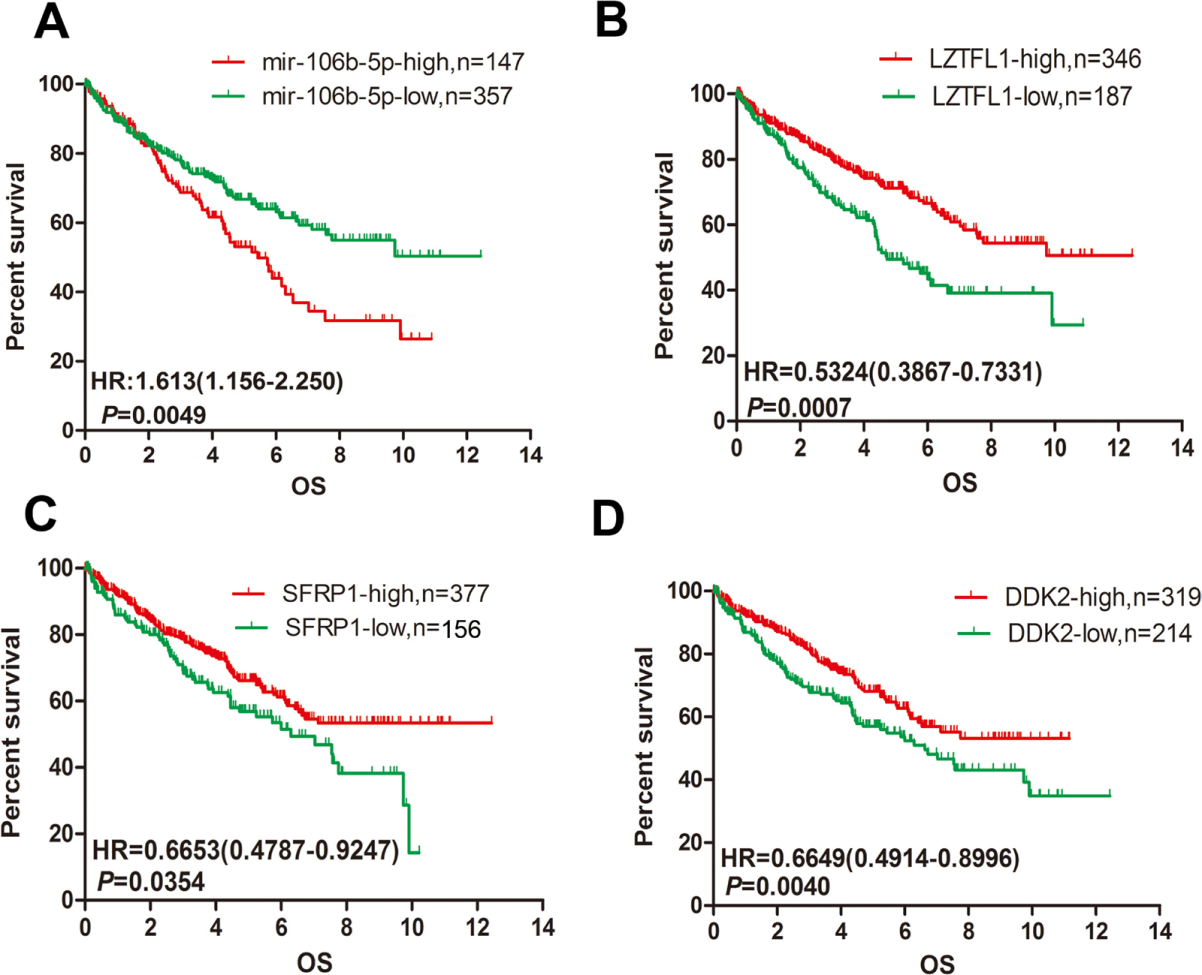
Upregulation of miR-106b-5p and downregulation of LZTFL1, SFRP1 and DKK2 is correlated with poor prognosis in ccRCC (**A**) Kaplan–Meier analysis of the correlation between the miR-106b-5p level and the OS of 504 patients with ccRCC in the TCGA cohort. (**B**–**D**) Kaplan–Meier analysis of the mRNA expression levels of LZTFL1, SFRP1 and DKK2 correlated with the OS of 533 patients with ccRCC in the TCGA cohort.

We analyse the prognostic significance of miR-106b-5p, LZTFL1, SFRP1 and DKK2 by using the ccRCC data set from TCGA to confirm their clinical prognostic value. There were 504 ccRCC patients with miRNA expression data and 533 patients with mRNA expression data in TCGA. We used X-tile program to obtain the optimal cut-point value. As we shown in Figure 6A, high expression of miR-106b-5p predicts poor overall survival (OS) in ccRCC patients by Kaplan–Meier survival analysis (log-rank test, *P* = 0.0049). Furthermore, the high mRNA expression level of LZTFL1, SFRP1 or DKK2 predicts better OS in 533 ccRCC patients (Figure 6B–6D).

## DISCUSSION

Recent studies revealed that Wnt signaling play roles in the development of ccRCC [20–23]. To elucidate the regulation of Wnt signaling transduction in ccRCC may contribute to the discovery of new therapeutic targets. Our data suggest that miR-106b-5p overexpression activates Wnt signalling in ccRCC through suppressing three Wnt antagonists and promotes ccRCC aggressiveness and stem-cell-like phenotype.

The most important downstream molecules of Wnt signaling in ccRCC are β-catenin and TCF [24]. Cytoplasmic accumulation of β -catenin was demonstrated to be associated with adverse clinical pathology and predicted poor survival in ccRCC patients [25]. Dysregulation of Wnt signalling and changes x of TCF4 binding are reported to be related to ccRCC progression [26]. In accordance with this observation, we found that β-catenin or TCF4 knockdown by siRNA transfection directly repressed Wnt/β-catenin signalling, eliminated the enhancement of miR-106b-5p overexpression on ccRCC cells stemness and tumorigenesis. Our results suggested that miR-106b-5p mediated activation of Wnt/β-catenin/TCF signalling contribute to the development of ccRCC.

Downregulation of members of SFRP family and DKK family [10, 11, 23] has been reported to correlate with the loss of function of Wnt antagonists in ccRCC. Significant decrease in tumorigenic ability in nude mice was observed in SFRP1-silenced ccRCC cells [27]. The mRNA and protein expression level of DKK was significant decrease in ccRCC patients [28]. LZTFL1 regulates the nuclear translocation of β-catenin and correlates with Wnt signaling [29–31]. Our data shown LZTFL1, SFRP1 and DKK2 were simultaneously suppressed by miR-106b-5p, which might be help to the explanation of the loss-of-function of Wnt antagonists in ccRCC. Furthermore, the data from TCGA shown the low expression of LZTFL1, SFRP1 or DKK2 in ccRCC correlates with poor OS, which indicating the important role of the three Wnt inhibitors in ccRCC progression.

miRNAs might be potential therapeutic targets for their roles in regulating many tumor suppressor genes [32–34]. miR-106b has been shown to function as an oncogene in various tumor types by regulating the proliferation and invasion of cancer cell [15, 16, 35–37]. In our study, the role of miR-106b-5p in promoting stem cell-like properties of ccRCC cells was focused. Similar roles of miR-106b in cancer stem cell characteristics were seen in gastric cancer [36]. Because of the important role of Wnt signaling in CSCs and the selective targeting of CSCs to reduce tumorigenesis of cancers, there are numerous studies worldwide to develop effective inhibitors of Wnt signaling [38]. In our study, silencing of miR-106b-5p leaded to Wnt signalling inactivation and significantly inhibited renal CSCs and tumorigenesis. These results demonstrate that miR-106b-5p might be a potential therapeutic target for Wnt signalling in ccRCC to suppress tumorigenesis.

Our current study suggested that overexpression of miR-106b-5p in ccRCC cells activates Wnt signalling by simultaneous suppression of LZTFL1, SFRP1, and DKK2 and constitutive promoting CSCs and tumorigenesis in ccRCC. Meanwhile, miR-106b-5p silenced in ccRCC cells leaded to inactivation of Wnt signalling and suppression of ccRCC cells stemness and tumorigenesis. Our data indicate that miR-106b-5p might be an important therapeutic target for Wnt signalling in ccRCC cells stemness to suppress tumorigenesis.

## MATERIALS AND METHODS

### Cell lines and clinical samples

769-P, 786-0, A498, ACHN, HK-2, Caki-1, Caki-2 were bought from American Type Culture Collection and were maintained in appropriate medium. OSRC-2 cell was bought from bought from National Platform of Experimental Cell Resources for Sci-Tech (Wuhan, China) and was maintained in T -medium supplemented with 5% FBS. A panel of 20 fresh ccRCC tissues and matched adjacent non-tumor normal tissues were collected from 20 patients with ccRCC treated at the First Affiliated Hospital of SYSU between January 2014 and August 2014 and stored in liquid nitrogen until further use. Written informed consent was obtained from all patients before the study. This study was approved by Ethical Committee of Sun Yat-sen University Cancer Center (Guangzhou, China).

### RNA isolation and quantitative RT-PCR

Total miRNA was isolated from cultured cells and surgically resected fresh ccRCC tissues using a mirVana miRNA Isolation Kit (Ambion, Austin, TX). The miRNA levels were assayed with Taqman probes and primer sets (Applied Biosystems, Foster City, CA, USA) in accordance with the manufacturer's instructions. Bulge-loop™ miRNA qRT-PCR Primer Sets (one RT primer and a pair of qPCR primers for each set) specific for miR-106b-5p are designed by RiboBio (Guangzhou, China). Extraction of total RNA and measurement of mRNA quantity were performed as described previously [32]. RNA was extracted from cells using TRIzol (Invitrogen) following protocols supplied by the manufacturer. First-strand cDNA was generated by MMLV transcriptase (Promega) using random primers. Real-time RT–PCR was performed on a CFX96 real-time PCR detection system (Bio-Rad), and a Roche SYBR FAST Universal qPCR Kit (RocheMolecular Biochemicals) was used for gene detection. The remaining qPCR primers are listed in Supplementary Table 1)

### Vector construction and lentivirus packaging and transduction

The miR-106b-5p expression vector, miR-106b-5p inhibitor vector and control vector for miR-106b-5p were purchased from GenePharma (Suzhou, China). miRNA sponges are transcripts with repeated antisense miRNA sequences that can sequester endogenous miRNAs as targets [39]. The sequence used to construct a lentiviral vector was as following: miR-106b-5p mimics: 5′-TAAAGTGCTGACAGTGCAGAT-3′; miR-106b-5p inhibitor sponge: 5′-ATCTGCACTGTCAGCACTTTA-3′; Control sequence: 5′-TTCTCCGAACGTGTCACGT-3′. The 3′-UTRs of LZTFL1, SFRP1 and DKK2 were amplified and cloned downstream of the luciferase gene in a modified pGL3 control vector (Promega). Vectors were packaged in 293T cells using ViraPower Mix (GeneCopoeia, Guangzhou, China). After culturing for 48 h, lentiviral particles in the supernatant were harvested and filtered by centrifugation at 500 × g for 10 min, and then transfected into ccRCC cells. The cells were cultured under puromycin (2 μg/ml) selection for 2 weeks, at which point real-time PCR was used to determine the level of miR-106b-5p. β-catenin-siRNA and TCF4-siRNA oligonucleotides and their corresponding control oligonucleotides were purchased from RiboBio (Guangzhou, China). Transfection of oligonucleotides was performed using Lipofectamine 2000 reagent (Invitrogen) according to the manufacturer's instructions.

### Western blot

For western blots, total cellular protein was extracted from cells and separated by sodium dodecyl sulfate–polyacrylamide gel electrophoresis. Nuclear extracts were prepared using the Nuclear Extraction Kit (ActiveMotif), according to the manufacturer's instructions. The following antibodies were used: anti-β-catenin (Millipore, Billerica, MA), anti-LZTFL1 (Sigma, St. Louis, MO), anti-SFRP1 (Abcam), anti-DKK2 (GeneTex, San Antonio, TX), anti-HDAC1 (Santa Cruz Biotechnology, Santa Cruz, CA), anti-GAPDH rabbit monoclonal antibody (Sigma, St. Louis, MO).

### Sphere formation assay

Five hundred ccRCC cells were seeded onto 24-well polyHEMA-coated plates (Sigma–Aldrich, St Louis, MO, USA) and cultured for 2 weeks in DMEM/F12 medium (Invitrogen, Carlsbad, CA) supplemented with 4 μg/ml insulin (BIOIND, Kibbutz Beit Haemek, Israel), B27 (1:50, Gibco, Grand Island, NY, USA), 20ng/ml epidermal growth factor (Pepro Tech, Rocky Hill, NJ, USA), and 10ng/ml basic fibroblast growth factor (Pepro Tech, Rocky Hill, NJ, USA).

### Flow cytometric analysis

Cells were digested with 0.25% trypsin and resuspended in DMEM containing 2% fetal bovine serum at a concentration of 1 × 10^6^ cells/ml, and then preincubated at 37°C for 30 min with or without 100 μM verapamil (Sigma–Aldrich) to inhibit ABC transporters. The DNA-binding dye Hoechst 33342 (Sigma–Aldrich) was then added at a final concentration of 5 mg/ml and the samples were incubated for 90 min in the dark with periodic mixing. Finally, the cells were incubated on ice for 10 min and washed with ice-cold phosphate-buffered saline before flow cytometric analysis. The data was analyzed using Summit 5.2 software (Beckman Coulter).

### Luciferase reporter assay

Cells were seeded in triplicate in 24-well plates and allowed to settle for 24 h. The indicated plasmids plus 1 ng of pRL-TK Renilla plasmid were transfected into the cells using Lipofectamine 2000 reagent (Invitrogen). Forty-eight hours after transfection, dual-luciferase reporter assays by determining the enzyme activities of luciferase using a BioTek Synergy2 microplate reader (Bio-Tek, Winooski, VT) at wave lengths of 560 and 465 nm following the manufacturer's instructions (Promega).

### *In vivo* experiments

All experimental procedures were approved by the Institutional Animal Care and Use Committee of SYSU. BALB/c nude mice (4–6 weeks of age, 18–20 g, male) were randomly divided into 3 groups (*n* = 5 per group). The indicated cells were mixed with Matrigel (BD Pharmingen, Carlsbad,CA) (1:1 v/v) and implanted into renal capsules of the mice at three doses (3 × 10^6^, 3 × 10^5^, 3 × 10^4^). The mice were anesthetized and killed 45 days after inoculation, and the tumours were removed and sectioned. In the tumour metastasis analysis, ten 4-week-old BALB/c nude mice in each experimental group were injected with A498-miR-106b-5p or A498-vector cells, respectively. Briefly, 2 × 10^5^ cells were injected intravenously through the tail vein into each mouse in a laminar flow cabinet. Six weeks after injection, the mice were sacrificed and examined.

### TCGA data

For the TCGA set, clinical data, miRNA expression and mRNA expression (level 3 data, RNA-seq Version 2 Illumina) were downloaded from the TCGA data portal (http://tcga-data.nci.nih.gov/tcga/) on Jun 1, 2016. The clinical data included 537 retrospectively identified patients who underwent radical or partial nephrectomy between 1998 and 2010 for sporadic ccRCC. Of the 537 patients, miRNA expression data were available for 504 patients and mRNA expression data were available for 533 patients.

### Statistical analysis

The Mann–Whitney *U* test was used for comparing expression levels in malignant and non-malignant samples, and the Wilcoxon test was used for pairwise comparison Comparisons between groups were performed using Student's *t*-test. All error bars represent the mean ± s.e.m. derived from three independent experiments. For survival analysis, the Kaplan–Meier method was used, and the Log-rank test was used for comparing cumulative survival. Statistical analyses were carried out using IBM SPSS Statistics 20.0 (IBM, Armonk, NY). Statistical significance was set at 0.05.

## SUPPLEMENTARY MATERIALS TABLES


