# Supplementary material for: miR-106b-5p promotes renal cell carcinoma aggressiveness and stem-cell-like phenotype by activating Wnt/β-catenin signalling
Source: Oncotarget. 2017 Feb 21;8(13):21461–71. doi: 10.18632/oncotarget.15591 (PMC5400598; doi:10.18632/oncotarget.15591)
Supplement: Supplementary file 1 [file oncotarget-08-21461-s001.pdf]

miR-106b-5p promotes renal cell carcinoma aggressiveness and stem-cell-like phenotype by activating Wnt/β-catenin signalling

Supplementary Materials

Supplementary Table 1: The sequences of primers used in Real-time RT–PCR assay

Gene Sympol	Real-time-primer-up	Real-time-primer-down
SOX2	GCCGAGTGGAAACTTTTGTCG	GGCAGCGTGTACTTATCCTTCT
OCT4	ATTCAGCCAAACGACCATCT	TCTCACTCGGTTCTCGATACTG
ABCC2	CCGTATCAGGTTTGCCAGTT	TGGAGGTGATCCAGGAAAAG
CXCR4	ACTACACCGAGGAAATGGGCT	CCCACAATGCCAGTTAAGAAGA
CD105	CGCACCGATCCAGACCACTC	CCCGGCTCGATGGTGTTGGA
TWIST1	TCCATTTTCTCCTTCTCTGGAA	CCTTCTCGGTCTGGAGGAT
MYC	CACCGAGTCGTAGTCGAGGT	TTTCGGGTAGTGGAAAACCA
MMP7	GCATCTCCTTGAGTTTGGCT	GAGCTACAGTGGGAACAGGC
CCND1	GGCGGATTGGAAATGAACTT	TCCTCTCCAAAATGCCAGAG
CD44	CACGTGGAATACACCTGCAA	GACAAGTTTTGGTGGCACG
BMP4	GCATTCGGTTACCAGGAATC	TGAGCCTTTCCAGCAAGTTT
FGF18	CTTACGGCTCACATCGTCC	ACTTCCTGCTGCTGTGCTTC
LZTFL1	AGAGTTGGGCCTAAATGAGCA	CACAGCTTGTAATCCATTGAGGA
SFRP1	GGCTTCTTCTTCTTGGGGAC	ATCTCTGTGCCAGCGAGTTT
DKK2	CTCACAGATCGGCAGTTCG	ATGCCAGTCCTTGGTACATGC
GAPDH	CTCCTCCACCTTTGACGCT	GGGTCTCTCTCTTCCTCTTGTG
